# Modelling Well-Being with Mindfulness Intervention on Bottom- and Middle-40% Income Earners in Malaysia

**DOI:** 10.3390/ijerph20043480

**Published:** 2023-02-16

**Authors:** Fatin Zaida Zaidi, Ming-Ming Lai, Anisah Jumaat, Yvonne Lee

**Affiliations:** Faculty of Management, Multimedia University, Cyberjaya 63100, Malaysia

**Keywords:** mindfulness, well-being, perceived stress, financial desire discrepancies, Malaysia

## Abstract

This paper examines mindfulness as a costless cognitive asset in reducing stress and improving subjective well-being and psychological well-being among Malaysian bottom-forty-percent and middle-forty-percent income earners, known as B40 and M40, respectively. The participants recruited for this experimental study were divided into intervention and control groups and completed pre- and post-assessment questionnaires. The leveraging on digital technologies during pandemic times from May to June 2021 enabled participants in the intervention group (n = 95) to undergo four weekly online mindfulness intervention sessions through Google Meet and completed daily home mindfulness practices using the mobile application for mindfulness: the MindFi version 3.8.0 mobile app. Based on the Wilcoxon signed-rank test, the intervention group’s mindfulness and well-being levels increased significantly after four weeks. This outcome contrasted to those in the control group (n = 31), who exhibited lower mindfulness and well-being levels. The PLS-SEM structural model consists of mindfulness as an independent variable, subjective and psychological well-being as dependent variables, and perceived stress and financial desire discrepancies as the mediators. This model has a goodness-of-fit of 0.076, proving that it is a fit and strong model. There is a positive relationship between mindfulness and subjective well-being (β = 0.162, *p*-value < 0.01). This model supports the mediation effect of perceived stress between mindfulness and subjective well-being variables (β = 0.152, *p*-value < 0.05). The overall structural model implies that the effectiveness of mindfulness intervention training not only enhanced bottom- and middle-income earners’ well-being but also lowered the perceived stress level that, henceforth, brought the mind and body together in the present moment.

## 1. Introduction

Malaysians were reported by the National Worry Index (NWI) to be extremely worried on the issues of the high cost of living, unaffordable housing, unemployment among the youth, and the increase in personal debts. This National Worry Index (NWI) reported an increment from 0.77 (in 2019) to 0.79 (in year 2020) [1]. Being in a state of worrying for too long leads to humans reacting negatively towards these financial stressors [2], and ironically leads to these individuals spending a lot and living beyond their means. As a result, people start spending as a reaction towards their financial worries and stress, mainly known as stress spending. This is due to stress and worry that causes people’s mind to wander, and they eventually will feel unhappy, as nearly 50% of people are not paying attention to what they are doing now and live in auto-pilot mode [3]. Naturally, humans’ bodies exist constantly in the present moment, and when they let their minds worry and wander, this leads to disharmony between the body and mind. As a result, people have more demands, consume more, and begin to live beyond their means.

Malaysians, especially low-income earners, live beyond their means and are more prone to financial stress as proven by Loke et al. [4], where about 61.5% of the low-income earners from the study had outstanding loans with the bank and could not even afford their living expenses. The Malaysia Credit Counseling and Debt Management Agency (AKPK) reported that Malaysians’ financial stress in 2020 increased by around 35%, especially since the COVID-19 virus outbreak began. AKPK indicates that high living expenses and the COVID-19 pandemic cause higher stress and worry levels among Malaysians [5], which is supported by Mahdzan et al. [6], who reported that bottom forty percent (B40) Malaysians who earned less than MYR 4849 monthly and the middle forty percent (M40) who earned in between MYR 4850 and MYR 1096 [7] are prone to higher financial stress levels and are looking for debt moratorium and lengthened repayment options. These statistics highlight the stark fact that bottom-forty-percent and middle-forty-percent income earners in Malaysia, known as the B40 and M40 groups, are struggling to meet their needs.

It is observed that people tend to have more desires and consume more when they are stressed and unhappy. People will shop a lot because people believe stress spending will make them calm, but this only works on a short-term basis [8]. Having more money or a higher salary does not necessarily improve one’s well-being [9,10]. There is a general belief that a higher income level would bring them happiness and better well-being, but this is not necessarily true. This is due to a lack of understanding of the strategies to effectively address financial inadequacies without spending additional financial resources. Practicing mindfulness does not require anyone to spend further to purchase equipment towards improving one’s well-being, as mindfulness is a cognitive asset all humans possess (In the context of this study, participants were equipped with free MindFi version 3.8.0 mobile app subscription once he or she was agreed to participate in this mindfulness program, and the program itself was provided at no charge.). Mindfulness practices can be performed either through formal practice, i.e., taking deep breaths while sitting down, or through informal practice, i.e., focusing on chewing and swallowing food during mealtimes without watching television or scrolling through one’s phone. Practicing mindfulness can increase an individual’s attention and, thus, lead to an increased feeling of happiness. Instead of making individuals wealthy in a monetary sense, mindfulness helps them become more aware of their needs and wants, enabling them to make wiser financial decisions. Mindfulness practices can reduce the overwhelming feeling of the financial decision of either using credit cards to purchase needed groceries or maxing out credit cards for unnecessary things such as new office outfits. Hence, financial stress does not equate to financial problems, but is related to the myriad of financial choices that need to be made [11].

Initially, the term “mindfulness” was taken from the core concept of Buddha teachings, namely the Pali word “sati”, which means “to remember”. An American, Kabat-Zinn, conceptualized mindfulness practice that is secular and free from any religious sentiments [12]. With the knowledge acquired from Buddhist meditation masters, he created the fundamentals of mindfulness concepts and practices for mainstream audiences. He pointed out that focus and awareness of the present moment are the fundamentals of practicing mindfulness. Mindfulness in this context is defined as “bringing one’s complete attention to the experiences occurring in the present moment in a non-judgmental or accepting way” [13].

People who regularly practice mindfulness, either through formal practices such as sitting and performing mindful breathing exercises or through informal practices such as being focused while walking or eating, will eventually respond to life’s challenges better. Mindfulness practices change the location of our brain activity from the “usual” and “old” us to the “latest” part of our brain [14]. A mindless mind, i.e., the “usual” and “old” state of the brain, leads people to respond to life’s difficulties hastily and superficially. In contrast, mindfulness practices send better signals to the individual’s amygdala and prefrontal cortex, leading them to make better decisions and create the “new” and “latest” version of themselves and their brain activity.

There is currently a lack of studies examining mindfulness intervention’s effects on enhancing the subjective and psychological well-being of low-income earners who are more prone to face both financial stress and financial discrepancy. Thus, this extant study fills in the gaps by proposing that the well-being of Malaysian B40 and M40 income earners can be enhanced through mindfulness training. Mindfulness will not make people rich. Instead, it makes people be aware of what they need and want which, in turn, allows them to make better financial decisions. Mindfulness allows people to change their attitude toward stress by shifting their negative thoughts to positive thoughts, thus enabling them to tackle their stress differently. This study integrates mindfulness as the independent variable, together with subjective well-being and psychological well-being as the dependent variables. The perceived stress and financial desire discrepancies work as the mediators of mindfulness and well-being.

## 2. Materials and Methods

### 2.1. Literature

A mindfulness intervention program crafted through the application of mindfulness practices, named mindfulness stress-based reduction (MBSR), originally aimed to reduce the pain and stress faced by chronic illness patients [12]. MBSR was one of the earliest mindfulness interventions programs and has become a benchmark for mindfulness research. MBSR programs last 8 to 10 weeks, where the participants and the trainer meet for 2 to 2 and a half hours weekly. The program includes homework assignments and lessons on practicing mindfulness during the weekly sessions.

Depending on their resources, humans may perceive a situation as stressful. Perceived stress was defined as “feelings or thoughts that an individual has about how much stress they are under at a given point in time” [15]. People become stressed out when they believe that certain circumstances are pushing them to exceed their resources and jeopardizing their well-being [16]. Mindfulness will lead to an increase in the hippocampus area and a decrease in the grey concentration of the amygdala [17,18], which makes the thinking brain (prefrontal cortex) receive more oxygen and better signals. Thus, people will worry less and respond to situations in a thoughtful manner [19,20]. Mindfulness practices’ impact on the human brain led researchers to implement mindfulness-based cognitive therapy. A systematic review performed by Chacko et al. [21] summarizes that mindfulness intervention programs significantly impact on the participants’ cognitive level and, thereafter, reduce stress.

Well-being serves as a measurement of one’s quality of life [22]. Well-being is divided into subjective well-being and psychological well-being. Subjective well-being is “the scientific term for happiness and life satisfaction—thinking and feeling that your life is going well, and not badly” [23]. Subjective well-being consists of life satisfaction, the presence of a positive mood, and the absence of a negative mood [23]. Life satisfaction is characterized as the overall evaluation of one’s life, a reflection of an individual’s judgement of their own life. An individual’s level of contentment with their life typically depends on how they feel. Maximizing “happiness” equates to maximizing one’s well-being [24]. Subjective well-being is mostly about people evaluating their lives based on emotional and cognitive abilities that they regard as happiness, peace, fulfilment, and life satisfaction [22]. On the other hand, Ryff [25] indicated that psychological well-being is “overall human contentment and functioning”. The six constructs of psychological well-being (autonomy, personal growth, self-acceptance, life purpose, mastery, and positive relations) were identified [26]. The primary goals of these six key constructs are to promote good emotional and physical health, and mainly positive feelings [26].

The effectiveness of mindfulness practices towards well-being has been explored and proven by Howells et al. [27], whereby 57 respondents who practiced mindfulness for 10 min daily using the Headspace mobile application had better mindfulness and well-being after ten days of intervention. On the other hand, 77 Malaysian undergraduate medical students who underwent a four-week mindfulness intervention demonstrated greater levels of mindfulness and psychological well-being after attending face-to-face training sessions for three hours each week and engaging in daily home practices for 15 to 20 min [28]. This example proves that the original eight-week duration of mindfulness intervention based on the MBSR program can be shortened but be just as impactful. The shortened program period was also proposed by Carmody and Baer [29], who highlighted that shorter period of mindfulness intervention successfully benefited the participants in increasing their mindfulness levels. To study the link between mindfulness and subjective well-being, the Mindfulness Attention and Awareness Scale (MAAS) [30] was created and utilized. The researchers found that respondents with high mindfulness scores reported high levels of subjective well-being [30]. Li et al. [31] also utilized the MAAS in a one-time survey on 627 adolescents who practiced mindfulness and reported that the participants had better subjective well-being levels.

Researchers analyzed the relationship between life satisfaction and perceived stress through a one-time questionnaire and reported that respondents with higher stress levels tend to have lower life satisfaction levels [32]. The degree to which situations are viewed as stressful is based on cognitive thinking. Perceived stress affects the brain through the hippocampus, which regulates emotions and overrides the amygdala to respond to threats or stress. At the same time, the central brain part, the prefrontal cortex, receives information and controls how humans react towards stressors [33]. The functioning of cognitive capacities can be influenced by practicing mindfulness. Baer et al. [34] discovered that four weeks of a mindfulness intervention helped in increasing the respondents’ levels of mindfulness, subsequently leading to a decrease in stress levels. Similarly, undergraduate psychology students who attended a brief mindfulness program for 6 weeks were found to have lower perceived stress levels [35]. All in all, past research showed that mindfulness practices could impact how one perceives and handles stress. Stress is also manifested when there exists a “gap between current financial and desired states” or a financial desire discrepancy [36]. According to the multiple discrepancies theory, by comparing what others have, what one has had in the past, and what one expects to have in one’s life, the existence of a perceived “gap” amongst these three elements will affect one’s life satisfaction [36]. A one-time survey by Crawford Solberg et al. [37] indicated that the respondents, who were University of Illinois students that kept comparing their financial status with their past and with others’ statuses, had a lower level of happiness due to them being overwhelmed by desires and not feeling satisfied with their life. Similarly, a study by Brown et al. [30] suggested that mindfulness encourages the mindset of having “enough”, as the respondents with strong financial desires could accept their current financial situation after practicing mindfulness.

### 2.2. Hypotheses

**Figure 1:**
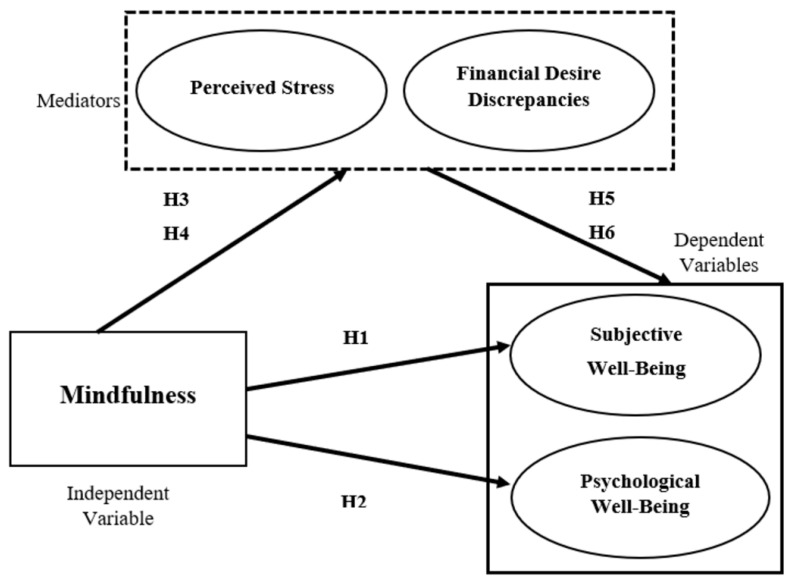
Conceptual Framework.

The above literature review provided insights into mindfulness, subjective well-being, and psychological well-being. It is believed that mindfulness has a positive relationship with subjective and psychological well-being, which is mediated by perceived stress and financial desire discrepancies. The conceptual framework showing the linkages of all the variables is presented in Figure 1. Six hypotheses have been developed to examine the relationship between mindfulness and well-being and the mediation effect of perceived stress and financial desire discrepancies. The proposed hypotheses are as follows:

**H1.**  *There is a positive relationship between mindfulness and subjective well-being among Malaysian B40 and M40 income earners*.

**H2.**  *There is a positive relationship between mindfulness and psychological well-being among Malaysian B40 and M40 income earners.*

**H3.**  *There is a mediation effect of perceived stress between mindfulness and subjective well-being among Malaysian B40 and M40 income earners*.

**H4.**  *There is a mediation effect of perceived stress between mindfulness and psychological well-being among Malaysian B40 and M40 income earners*.

**H5.**  *There is a mediation effect of financial desire discrepancies between mindfulness and subjective well-being among Malaysian B40 and M40 income earners*.

**H6.**  *There is a mediation effect of financial desire discrepancies between mindfulness and psychological well-being among Malaysian B40 and M40 income earners*.

### 2.3. Participants

The researchers took into account the following criteria in recruiting and handling participants: (1) the interest of researchers; (2) group allocation and blinding participants and administrators; and (3) the appropriate method for intervention.

Firstly, the interest of researchers in recruiting participants was based on the inclusion criteria. The participant inclusion criteria were as follows: (1) they must be Malaysian B40 and M40 income earners with a monthly income level of less than MYR 10,959; (2) they must be able to understand English; and (3) they must own a smartphone. Based on the inclusion criteria, the nonprobability sampling method of purposive sampling approach was used. This allowed the researcher to have sample specificity in choosing participants that were relevant to the study [38]. This purposive sampling approach was based on the type, nature, and objective of this study, whereby it only included people who were appropriate for the study’s objectives and the participants were selected based on them being in the B40 and M40 income groups. This nonrandom technique does not need underlying theories or a set number of participants. By deciding what needs to be known, the researchers identified people who could and were willing to participate and were able to provide the information on a phenomenon of interest [39], focusing on the effect of mindfulness on the well-being of B40 and M40 income earners. The participants were recruited from April until mid-May 2021 using a purposive sampling approach, which is in parallel with studies by Alimehdi et al. [40] and Sa’id and Dewi [41].

Secondly, the participants for this researcher’s experimental study were divided into two groups: an intervention group and a control group. The classification and allocation of the number of participants in the two groups were based on whether they could attend four weekly webinars. Thus, the participants indicated their willingness by clicking on the registration form based on these two options: option 1—“I am willing to attend four live weekly live webinars and answer surveys” or option 2—“I am willing to answer surveys only and not able to attend four live weekly webinars”. The researchers considered the willingness of the participants as the live weekly webinars were held for four consecutive weekends on Saturdays. From the responses, the researcher allocated the participants who clicked option 1 into the intervention group and those who clicked option 2 into the control group. All the participants were not aware that their responses would lead them to be allocated and classified into the treatment or control group and they were also unaware of the presence of two groups in the study. Researchers did not inform the participants about the presence of the intervention and control group in order to reduce the bias. Furthermore, the trainer for the corporate wellness program was not aware of the classification of participants into two groups: the intervention and control groups. The trainer only focused on training the participants who attended the four weekly live webinars. Additionally, to prevent bias and protect the privacy of all the participants, the researchers did not disclose any information of the participants to the trainer. Following this, the trainer was totally unaware of the presence of the control group throughout the study.

Lastly, the method of intervention for this study was entirely remote due to the COVID-19 pandemic situation in Malaysia. The corporate wellness program was held from 22 May 2021 to 12 June 2021, which was at the same time the movement control order (MCO) was enforced. During the MCO, citizens were prohibited to cross states’ borders and face-to-face meetings were not allowed either [42]. The participants and the researchers were living in different states in Malaysia and an online webinar was the most appropriate method to conduct the intervention. In addition, the trainer, Mr. Bjorn, the CEO and co-founder of MindFi, is a Singaporean residing in Singapore. With the rules of the MCO from the Malaysian government that led to international border closures, this restricted him from travelling to Malaysia and conducting face-to-face mindfulness intervention. During week 3 and week 4 of the corporate wellness program, starting 1 June 2021, the government of Malaysia enforced total lockdown, whereby all sectors were not allowed to operate during this period except for essential economic and service sectors, and all citizens were required to stay at home [43]. Thus, the researchers believed that the online live webinars for four weeks were the best decision for conducting a mindfulness intervention and proceeded with this approach. Meanwhile, attention to the control group was given through regular engagement with them, as they did not have access to the online live weekly webinars, the video recording of these live webinars, or the MindFi version 3.8.0 mobile app.

Based on the three criteria listed, a modified randomized controlled trial (modified RCT) was thoroughly performed for this study. This approach is almost similar to conventional RCTs, except for the full randomization and allocation among the participants that were modified to suit the pandemic situation which led to mobility restrictions due to movement control orders in Malaysia.

**Figure 2:**
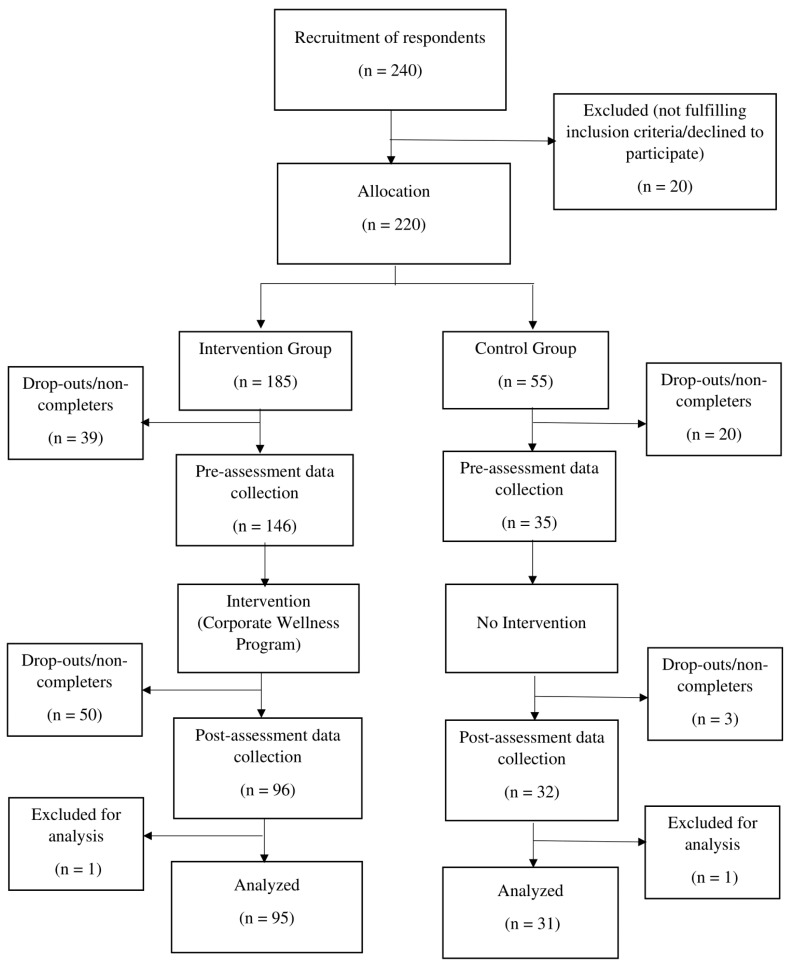
Participant recruitment process flow diagram.

In May 2021, there were 240 respondents signed up for the corporate wellness program, and based on the inclusion criteria, 220 respondents were allocated for the program. The recruitment poster is attached in Appendix A. There were originally 185 participants in the intervention group and 55 respondents in the control group. The drop-out participants and noncompleters of both questionnaires were excluded from the analysis. The data from ninety-five participants and thirty-one respondents from the intervention group and control group, respectively, were analyzed. The recruitment process flow diagram is presented in Figure 2.

### 2.4. Interventions

The experimental study’s participants were required to answer two sets of questionnaires—pre-assessment and post-assessment questionnaires—which quantitatively measured the participants’ state of mindfulness, financial desire discrepancy, perceived stress, and well-being. Those who participated in the mindfulness intervention program were required to answer the pre-assessment questionnaire and attend a mindfulness intervention program, named a corporate wellness program, held from 22 May to 12 June 2021. Throughout the four weeks of the corporate wellness program, the participants attended weekly online webinars every Saturday from 11.30 a.m. to 1 p.m., where the participants were guided by Mr. Bjorn Lee, a mindfulness practitioner and skilled trainer, who is also the CEO and cofounder of the MindFi mobile app. The participants of the corporate wellness program were given access to the video recordings of the weekly online webinars, which they referred to for performing the required daily mindfulness practices for 10 min using the MindFi version 3.8.0 mobile app, which helped guide the users on their meditation practices. After four weeks of the corporate wellness program, the participants were required to answer the post-assessment questionnaire. In addition, the participants were awarded with an e-certificate for their diligent participation throughout the four weeks of the corporate wellness program.

Within the same timeline, respondents in the control group were required to answer identical questionnaires (namely, the pre- and post-assessment questionnaires) to the intervention group without attending and being involved in the mindfulness intervention program. The respondents in the control group did not have access to the online weekly webinars, the video recording of those webinars, or the MindFi mobile app. The control group was used to compare the outcomes of the mindfulness intervention group to those of the control group and to identify any differences in outcomes.

The details and activities of the mindfulness intervention program are presented in Table 1. Before the actual data collection, a pilot study was conducted in April 2021 among 32 respondents who were B40 and M40 income earners. The questionnaires’ items from the pilot study were tested. For actual data collection, some items were added and removed based on the pilot test’s reliability analysis. The items used to measure each variable were adopted from past studies and measured using the Likert-scale form, except for demographic questions.

### 2.5. Measurements

The independent variable, mindfulness, was assessed using construct attention and awareness. For the dependent variables, both subjective and psychological well-being variables were used to measure the respondents’ state of well-being. The subjective well-being variable was divided into three constructs, which were life satisfaction, positive affect, and negative affect. The psychological well-being variable consisted of six constructs—autonomy, personal growth, self-acceptance, life purpose, environmental mastery, and positive relation. The variables used to assess the mediators for this present study were the perceived stress and financial desire discrepancies. The financial desire discrepancies were divided into three constructs, which were current financial desire discrepancy, past desire comparison, and social desire comparison.

To measure the mindfulness level of the respondents, this study adopted a 15-item question from the Mindful Attention Awareness Scale (MAAS) [44]. Based on past research, this scale has strong reliability (r = 0.81) and validity [30,45]. On a scale from 0 to 6, this MAAS scale gauges respondents’ attention and awareness (from “almost always” to “always never”). There are three constructs for subjective well-being, which are life satisfaction, positive affect, and negative affect. The Satisfaction with Life Scale (SWLS), developed by Diener et al. [46], was adopted to measure life satisfaction. This scale measures one’s overall life satisfaction and consists of 5 questions with Likert scales from 1 to 7 [27,31,47]. This measure has been shown to have excellent validity and reliability (r = 0.82). The remaining constructs of subjective well-being, the positive affect and negative affect, were adapted from Thompson’s study [48]. I-PANAS-SF has 10 items, each with 5 questions about positive affect and 5 about negative affect, and the scale was used as the measurement to examine subjective well-being [49,50].

Psychological well-being is measured through the Psychological Well-Being Scale created by Ryff [26]. The PWB scale has six constructs, which are autonomy, personal growth, self-acceptance, life purpose, environmental mastery, and positive relation. Good test–retest reliability and high validity have been shown for this PWB scale [51]. This study adopted all 18 items, listed in Appendix B, which respondents answered on a Likert scale from 1 to 7. The mediators were the perceived stress and financial desire discrepancies. The Perceived Stress Scale (PSS) was used to examine how perceived stress affected the respondents through a 10 Likert scale from 0 to 5 that asked respondents about their emotions and thoughts [52]. The Financial Desire Discrepancies Scale (FDD scale) was adopted by Brown et al. [30] whereby the respondents needed to answer six questions on their wants vs. their needs based on three constructs (current financial desire discrepancy, past desire comparison, and social desire comparison), on Likert scales of 1 to 7 or 1 to 8.

The internal consistency for all the scales used for both assessments was calculated using Cronbach’s alpha coefficient and reported to have good internal reliability and consistency. Reliability analysis can be found in Section 3.

### 2.6. Statistical Analysis

The data gathered from pre- and post-assessment questionnaires from both groups were screened and assessed using the Statistical Package for the Social Sciences (SPSS) version 26. Firstly, the Wilcoxon signed-rank test was used to analyze the effectiveness of mindfulness intervention on the participants of the corporate wellness program. By comparing the scoring from pre- and post-assessments and the *p*-values, the effectiveness of the corporate wellness program could be seen in the participants’ well-being, stress, and financial desire discrepancies levels. In the same vein, the respondents in the control group were also assessed via the Wilcoxon signed-rank test through pre- and post-questionnaire comparison and the *p*-values. The comparison between the intervention and control groups was analyzed using the results of the Wilcoxon signed-rank test and descriptive statistics.

The next statistical analysis used was a structured model with the aid of the partial least squares–structural equation modelling (PLS-SEM) software SmartPLS 3. To evaluate the relationship between mindfulness as well as subjective and psychological well-being, selected items from the questionnaire were chosen in building a structured model that was then analyzed using structural equation modelling to determine its goodness-of-fit. In order to test the effectiveness of mindfulness on subjective and psychological well-being, PLS-SEM was used for this study as it is widely known to study relationships between the variables in this model through the testing of hypotheses. This is because it makes no assumptions about the distribution of the variables or the sampling distribution [51]. The mediation effect of perceived stress and financial desire discrepancies were also analyzed using the specific indirect effect analysis feature in PLS-SEM, following the advice from Hair et al. [51] that researchers should instead bootstrap the sampling distribution of the indirect impact to examine the mediating effects.

## 3. Results

The usable sample size from both groups was based on the completion of both assessments. There were 95 participants for the intervention group and 31 respondents for the control group. Table 2 shows the demographic profiles for both groups. For the intervention group, there were 95 participants, with 68 female respondents (71.58%) and 27 male respondents (28.42%). The participants were between 18 and 25 (44.2%), while those aged 35 to 45 accounted for 25.26%. About 20% and 9.53% of the respondents were aged 26 to 35 and 46 to 60, respectively, and most of them (47 respondents) were from urban areas (64.21%).

The control group had a total of 31 respondents, with 28 female respondents (90.3%) and 3 male respondents (29.7%). About 90.3% of the respondents were 18 to 25 years old, followed by 9.7% of respondents aged between 26 and 35. Similar to the intervention group, most respondents from the control group lived in urban areas.

The final column of Table 2 shows the *p*-values obtained from the Mann–Whitney U test for continuous variables and on chi-square association for categorical variables. The *p*-values more than 0.05 indicate that the two groups are not similar and provide a fair judgement on the effects from the mindfulness intervention.

All the variables’ items used for measurement were reliable, with Cronbach’s alpha values that were greater than 0.6. One item from each construct of psychological well-being was removed to improve the Cronbach’s alpha value. Additionally, one of the psychological well-being constructs (purpose in life) was removed entirely. The list of items removed is listed in Appendix C. The results of the reliability analysis are reported in Table 3. The remaining questionnaire items were then assessed and compared to determine the effectiveness of mindfulness on the variables of subjective well-being, psychological well-being, perceived stress, and financial desire discrepancies.

### 3.1. Wilcoxon Signed-Rank Test

The effectiveness of mindfulness intervention on the respondents was analyzed using the nonparametric test. As the questionnaire items were in Likert scale form and the data were not normally distributed based on the normality test, the Wilcoxon signed-rank test was used to determine the effectiveness of mindfulness intervention on the subjective and psychological well-being level of the respondents.

The subjective well-being level of the respondents in the intervention group was compared between the post-intervention and pre-intervention scores using the Wilcoxon signed-rank test. According to the mean value for the life satisfaction construct, the respondents’ life satisfaction improved post-assessment (M = 4.356, SD = 1.278) compared to pre-assessment (M = 4.023, SD = 1.383); (*t* = 2298.50, z = −2.526, *p*-value < 0.1). The construct of positive affect showed a significant difference between pre- and post-assessment values (*t* = 2320.50, z = −2.633, *p*-value < 0.1).

For psychological well-being, five constructs of psychological well-being were analyzed. The first construct was autonomy with post-assessment (M = 5.095, SD = 1.403) being higher than pre-assessment values (M = 4.684, SD = 1.502). The autonomy construct improved respondents’ psychological well-being post-assessment compared to pre-assessment (*t* = 1989.00, z = −2.246, *p*-value < 0.1). The remaining four constructs (environmental mastery, personal growth, positive relation, and self-acceptance) also showed significant differences post-assessment compared to pre-assessment with post-assessment values significantly higher than pre-assessment values (*p*-value < 0.1). These results show that the mindfulness intervention (corporate wellness program) was effective in improving respondents’ subjective and psychological well-being with a *p*-value < 0.1. Furthermore, the effectiveness of mindfulness practices was found to reduce the mediators’ variables whereby the perceived stress and financial desire discrepancy were significantly reduced, with z = −1.864, *p* = 0.031 and z = −2.2463.234, *p* = 0.001, respectively.

For the respondents from the control group, the subjective well-being level was also compared between the post-intervention and pre-intervention score conditions using the Wilcoxon signed-rank test. According to the mean value for the life satisfaction and positive affect construct, the respondents’ life satisfaction and positive affect showed minimal and not significant improvements (*p*-value > 0.05). This study posits that the absence of mindfulness intervention leads to insignificant changes in life satisfaction and positive affect (*p*-value > 0.05), together with a significant increase in the negative affect feelings among the control group respondents after four weeks. For psychological well-being, all five psychological well-being constructs (autonomy, environmental mastery, personal growth, positive relation, and self-acceptance) reported to have a reduction in their means, indicating an overall decline in the respondents’ psychological well-being level. The importance of the mindfulness intervention was proven in the nonsignificant *p*-values of the perceived stress and financial desire discrepancy mediating variables among the control group respondents. Table 4 summarizes the mean values and *p*-values obtained from the Wilcoxon signed-rank test.

Analysis indicates raised levels of well-being levels and lowered perceived stress and discrepancies in financial desires after four weeks of the corporate wellness program based on the Wilcoxon signed-rank test findings. Given that the participants in the intervention group were given access to the MindFi mobile app and weekly online mindfulness webinars, these tools helped the participants to develop better mindfulness. In comparison, the respondents in the control group reported lower mindfulness and psychological well-being levels, and there was an increase in perceived stress levels in the fourth week, demonstrating the rationale and effectiveness of mindfulness practices. This was indicated by the findings of insignificant *p*-values based on the Wilcoxon signed-rank test. The descriptive statistics based on mean values for both groups are presented in Table 5.

### 3.2. Structural Model Using PLS-SEM

In order to build a structural model, responses of the participants from the intervention group (n = 95) were selected, in parallel with Martínez-Rubio et al. [53] who selected all the related samples in building a structural equation model. Stuart et al. [54] indicated that the generalizability is only a data analysis assumption as the most important thing is the design approach choices (e.g., the sample and choice of measures and efforts to provide measures comparable across studies) that are feasible for the work of researchers. The first step before building a structural model is to check and address common method bias issues, as the respondents for this study responded to the same questionnaire over the same time. By analyzing the output of collinearity, which is the variance inflation factor (VIF) value in PLS-SEM as proposed by Kock [55], it was found that the single-source bias for this study was not a serious issue because the variables’ VIF values were less than 3.3. The evaluation of the model uses a two-step process: the measurement model and the structural model [56]. The measurement model must first be tested to ensure the instruments are valid and trustworthy [57]. The measurement model was evaluated to determine if the items for each variable satisfied the conditions with the average variance extracted value of more than 0.5, respectively, and the composite reliability value higher than 0.7, as presented in Table 6. As the model’s instruments were valid and trustworthy, the model was further analyzed in the following section.

Next, the structural model that was bootstrapped using 5000 sample re-samples [57] was evaluated. The path coefficients and *p*-values were analyzed, as suggested by Hahn and Ang [58] with derived hypotheses presented in Table 7, which shows the path coefficients and *p*-values for this study. Firstly, selected items were chosen to reflect the whole subjective well-being rather than being divided into three constructs. Based on the three items selected from the life satisfaction construct, the results indicated a significant and positive relationship between mindfulness and subjective well-being (β = 0.162, *p*-value < 0.1), therefore supporting (H1). Secondly, instead of testing each of the five constructs of psychological well-being, specific items were selected to reflect the entirety of psychological well-being. The path coefficient between mindfulness and psychological well-being was reported as positive, with β = 0.146, but not significant (*p*-value = 0.104), showing that an increase in mindfulness leads to an increase in psychological well-being. Next, the evaluation of the mediating role of perceived stress was tested after bootstrapping using the indirect effect, as suggested by Preacher and Hayes [59,60]. According to Table 7, there was a significant mediation effect by perceived stress on the relationship between mindfulness and subjective well-being (β = 0.152, *p*-value < 0.05) as well as a mediation effect by perceived stress on the relationship between mindfulness and psychological well-being (β = 0.202, *p*-value < 0.05). As shown in Table 7, H3 and H4 were, therefore, supported. On the other hand, there was a lack of a mediation effect by financial desire discrepancies on the relationship between mindfulness and subjective well-being, as well as the mediation of financial desire discrepancies on the relationship path between mindfulness and psychological well-being. The *p*-values for both paths were more than 0.1. This meant that H5 and H6 were not supported.

Making case-level predictions and using a 10-fold process, as suggested by Shmueli et al. [61], the PLS Predict output generated was then evaluated for the predictive relevance of the model built. Table 8 shows that all the items’ values of this study’s structural model (column 2) were lower than those of the LM (linear model) (column 3), except one item of “Pleased with how things turned out so far”. This shows that the model built had strong predictive power. Additionally, the final structural model showed a square root mean squared residuals (SRMR) value of 0.076, proving that this structural model had a good fit.

**Figure 3:**
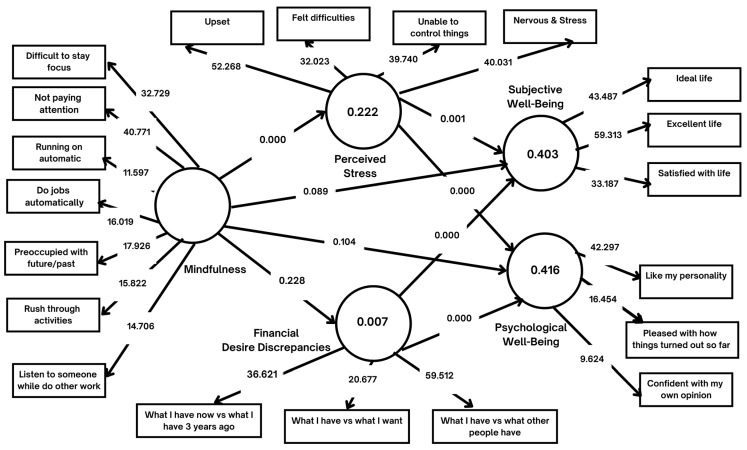
Structural Model.

Figure 3 shows the structural model, while Table 9 shows the summary of the hypotheses testing for this study based on the analyses above. There was an effectiveness in the mindfulness intervention (corporate wellness program), supported by the positive relationship (*p*-value < 0.1) between mindfulness and subjective well-being as well as between the former and psychological well-being. In addition, the role of perceived stress as a mediator between mindfulness and well-being was proven (*p*-value < 0.1).

## 4. Discussion

The structural model built depicted the linkage of mindfulness towards subjective and psychological well-being and the mediation effect of perceived stress and financial desire discrepancies. Firstly, four weeks of mindfulness intervention (the corporate wellness program) was proven adequate in boosting respondents’ mindfulness and well-being levels. Specifically, the respondents who attended 1.5 h of weekly webinars and carried out their own home daily practices of 10 min a day using the MindFi version 3.8.0 mobile app reported having better and higher mindfulness levels and well-being, besides also having a reduction in their stress and financial desire discrepancies levels. Secondly, mindfulness practices helped the respondents to be mindful and aware of the present moment. At the beginning of the mindfulness intervention program, the respondents reported that they lived in automatic mode, where they rushed through activities and had difficulty focusing while carrying out work. After four weeks of practicing mindfulness, they showed an increase in their mindfulness levels, whereby they could now focus and pay attention while carrying out their work and be in the present moment without being in automatic mode. From this, it can be depicted that mindfulness is not a form of breathing practice that helps people to feel calm instantly but can change one’s life to live in the present moment and indirectly increase one’s job productivity.

Furthermore, based on the items selected in building the structural model, the respondents’ well-being level increased, specifically life satisfaction. The respondents who were B40 and M40 income earners were initially unhappy and unsatisfied with their life due to their financial state, but now, they feel more satisfied after four weeks of mindfulness intervention. Mindfulness practices could “alter” the brain and the respondents’ view of their situation and current life, as they reported having an ideal and excellent life, despite having no changes in their income levels. On the other hand, for psychological well-being, it is undeniably quite challenging for a person to achieve better psychological well-being levels in a short time period as it comprises the whole view of one’s life towards emotional and physical health [26].

Despite having a positive relationship with mindfulness and an increase in mean values after four weeks of a mindfulness intervention, the structural model showed an insignificant *p*-value of slightly more than 10% for the relationship between mindfulness and psychological well-being. Although the participants in the intervention group were guided with various techniques of mindfulness practices through the corporate wellness program, the four-week program might need to be extended to a longer period to achieve a significant impact on psychological well-being. Given that the participants were new to mindfulness practices, perhaps a longer time for practicing mindfulness could help participants in understanding the concept and creating the habit of practicing mindfulness daily, which will then lead to higher psychological well-being levels.

Next, the findings depicted that the mindfulness intervention for four weeks was adequate in reducing stress levels among the participants, despite the enforcement of the movement control order during the COVID-19 pandemic in Malaysia in week 2 of this study. Indirectly, the movement control order made the respondents feel more stressed, especially without any guidance on handling stress. This is reflected by the increase in the perceived stress’s mean value among the control group in the fourth week of the assessment. Nevertheless, with mindfulness practices, the respondents in the intervention group had lower stress levels, proving that they can manage their stress by perceiving and selecting emotions wisely when they have the correct stress management technique, such as mindfulness. The mediation effect of perceived stress on respondents’ well-being was supported, demonstrating that consistent mindfulness practices help improve subjective and psychological well-being among B40 and M40 income earners through decreasing the perceived stress level. After four weeks of a mindfulness intervention, the respondents reported feeling less upset, nervous, and stressed and having fewer difficulties handling their stress levels. The exposure to mindfulness practices successfully influenced the participants in managing their stress levels, whereby the reduction in perceived stress increased participants’ subjective and psychological well-being.

Lastly, there was an observed effectiveness of four weeks of mindfulness intervention in decreasing financial desire discrepancies among the respondents, as can be seen from the reduction in the mean of the financial desire discrepancies score. Initially, B40 and M40 income earners used to feel vulnerable and worried about their low monthly income. Interestingly, after consistently practicing mindfulness for four weeks, they now report having lesser financial desires. Mindfulness practices helped the respondents make financial decisions as they reported having a narrower gap between what they have vs. what they want. Nevertheless, despite the lowering of financial desire discrepancies from mindfulness practices, the financial desire discrepancies variable itself was found not able to mediate the link between mindfulness and subjective well-being and psychological well-being. Considering that the participants were low-income earners in Malaysia, it was difficult to entirely remove their desire discrepancy, especially their tendency to make social and past financial comparisons. Humans are used to wanting and demanding more as we compare what we have now with others [36].

It requires a lot of time and effort to reduce one’s financial desire discrepancies, and this study suggests longer mindfulness intervention. As discussed, mindfulness practices are proven effective in reducing financial desire discrepancy based on the Wilcoxon signed-rank test. Hence, a slight change of adding more weeks to the mindfulness intervention program is suggested. The mediation effect of this lengthened mindfulness intervention program on financial desire discrepancies could then be studied.

This study contributes to the effectiveness of mindfulness intervention towards the well-being of Malaysian B40 and M40 income earners. The development of the structural model and analysis using the Wilcoxon signed-rank test indicated the effectiveness of these four weeks of mindfulness intervention. Future researchers may conduct a post-interview session to gain more information regarding the effectiveness and hindrance they experienced throughout the mindfulness intervention program. Additionally, it is suggested for future research to integrate mindfulness with minimalism so that people will tend to spend less and possess the things they genuinely love rather than buy more things. The results of this study would be a valuable source of information for enhancing well-being, especially for B40 and M40 income earners. Implementing mindfulness practices of simply focusing on the present moment and taking deep breaths could help reduce stress. The Ministry of Health could introduce mindfulness practices to its plan to boost well-being in creating better citizens. The Ministry of Health and government agencies in charge of society’s welfare can implement mindfulness practices to reduce stress and ease anxiety among lower- and middle-income earners in Malaysia.

## 5. Conclusions

Mindfulness can be trained to help bring the mind and body together in the present moment. The mindfulness and well-being levels of the bottom-40% and middle-40% income earners respondents increased after four weeks of intervention. They felt more satisfied with their life after four weeks of the mindfulness intervention program and were more mindful in handling their stress and financial desires. Hence, their stress and financial desire discrepancies levels reduced after the mindfulness intervention. This study adds to the evidence of using an online platform as a medium for mindfulness intervention programs via Google Meet and the mobile mindfulness application named MindFi. Stress can be managed with mindfulness, especially with the advancement of technology in the form of mindfulness mobile applications such as MindFi.

Through this study, it is proven that mindfulness intervention promotes well-being and reduces stress among low-income earners in Malaysia. The results of the Wilcoxon signed-rank test indicate that an increase in mindfulness level leads to better and significant subjective and psychological well-being. The stress level and financial desire discrepancies are also reduced. On the other hand, based on the structural model, mindfulness positively impacts subjective well-being (in particular, life satisfaction). There is a reduction in perceived stress due to mindfulness practices that eventually improve subjective and psychological well-being, through the significant effect of mediation on perceived stress. We believe longer mindfulness practices beyond four weeks will provide better evidence for the mediation effect of financial desire discrepancies. Shaping an individual is possible by implementing mindfulness interventions that boost their well-being. Therefore, individuals ought to continue to practice mindfulness to achieve a happier and more satisfying life, without additional financial resources or income being needed.

## Figures and Tables

**Table 1:** Details of Corporate Wellness Program (Mindfulness Intervention Program) from 22 May 2021 to 12 June 2021.

Session No. (Week)/Date	Module	Content	Practice
1 22 May 2021	Resilience and Stress Recovery	The concept of being in the present moment Building resilience using the pomodoro technique How to redeem the Mindfi mobile app premium free subscription	Five-finger breathing exercise Box breathing exercise (Navy seal breathing) Body and breathing sensation exercise
2 29 May 2021	Resilience and Stress Recovery	The impact of mindfulness on our brain and well-being The importance and benefits of gratitude Usage of the Mindfi mobile app	Five-finger breathing exercise Listening exercise (body and breathing sensation) Gratitude exercise
3 5 June 2021	Mental Energy and High Performance	Managing energy The benefits of practicing mindfulness in managing stress and burnout Usage of the Mindfi mobile app	Body scan and breathing sensation exercise Muscle relaxation exercise Mindful drinking exercise
4 12 June 2021	Mental Energy and High Performance	Concept of “Now” vs. “Yet” The benefits of practicing mindfulness and journaling Usage of the Mindfi mobile app	Body scan and breathing sensation exercise Question and answer session

**Table 2:** Baseline Characteristics of The Respondents.

Baseline Characteristics	Intervention Group	Control Group	*p*
M (SD) or %	n = 95	n = 31	
Demographics			
Gender			0.863
Female	68 (71.58)	28 (90.3)	
Male	27 (28.42)	3 (9.70)	
Age			0.076
18–25 years old	42 (44.21)	28 (90.3)	
26–35 years old	19 (20.00)	3 (9.70)	
36–45 years old	24 (25.26)	-	
46–60 years old	10 (10.53)	-	
State			0.919
Johor	5 (5.26)	3 (9.70)	
Kedah	-	1 (3.20)	
Kelantan	-	1 (3.20)	
Kuala Lumpur	9 (9.47)	6 (19.40)	
Malacca	13 (13.68)	-	
Negeri Sembilan	5 (5.26)	-	
Pahang	1 (1.05)	-	
Penang	2 (2.11)	1 (3.20)	
Perak	9 (9.47)	10 (32.30)	
Putrajaya	2 (2.11)	1 (3.20)	
Selangor	47 (49.47)	7 (22.60)	
Terengganu	2 (2.11)	1 (3.20)	
Residential Area			0.222
Rural	3 (3.20)	4 (12.900)	
Suburban	31 (32.6)	13 (41.90)	
Urban	61 (64.21)	14 (45.2)	
Highest Level of Education			0.255
Bachelor’s degree/professional degree	57 (60)	21 (67.70)	
Foundation level	-	1 (3.20)	
Master’s degree	19 (20)	1 (3.20)	
PhD	7 (7.37)	-	
Professional certificate	1 (1.05)	-	
Sijil Kemahiran Malaysia	1 (1.05)	-	
SPM/O Level Equivalent	6 (6.32)	1 (3.20)	
STPM/Diploma/A Level Equivalent	4 (4.21)	7 (22.60)	
Employment Status			0.897
Government sector	5 (5.26)	2 (6.5)	
Nonemployed	3 (3.16)	3 (9.7)	
Private sector	77 (81.05)	12 (38.7)	
Self-employed	-	4 (12.9)	
Student/still studying	10 (10.53)	10 (32.3)	
Self-Income			
Between MYR 4850 and MYR 10,959 (M40)	27 (28.42)	-	
Less than MYR 4849 (B40)	68 (71.58)	31 (100)	
Mindfulness			
Attention and awareness (MAAS)	4.01 (1.05)	3.77 (0.95)	0.201
Subjective Well-Being			
(i) Life satisfaction (SWLS)	4.02 (1.38)	3.76 (1.60)	0.403
(ii) Positive affect (I-PANAS-SF)	3.47 (0.79)	3.32 (0.71)	0.361
(iii) Negative affect (I-PANAS-SF)	2.42 (1.01)	2.64 (0.98)	0.371
Psychological Well-Being (Ryff’s PWBS)			
(i) Autonomy	4.69 (1.50)	5.27 (1.32)	0.048
(ii) Environmental mastery	4.96 (1.24)	5.29 (1.14)	0.171
(iii) Personal growth	6.07 (1.07)	6.31 (0.93)	0.333
(iv) Positive relation	4.24 (1.64)	4.00 (1.60)	0.524
(v) Self-acceptance	5.01 (1.36)	5.15 (1.39)	0.551
Perceived Stress (PSS)	2.05 (0.61)	2.11 (0.46)	0.621
Financial Desire Discrepancies (Brown’s FDD)			
(i) Desire discrepancies	4.38 (1.44)	4.60 (1.05)	0.501
(ii) Social desire discrepancy	3.97 (1.46)	4.19 (1.18)	0.274
(iii) Comparison desire discrepancy	3.24 (1.36)	3.69 (1.23)	0.086

**Table 3:** Reliability analysis.

Variables	No. of Items	Cronbach’s Alpha	
		Pre-Assessment	Post-Assessment
1. Mindfulness (MAAS)	15	0.942	0.940
2. Subjective Well-Being			
(i) Life satisfaction (SWLS)	5	0.879	0.880
(ii) Positive affect (I-PANAS-SF)	5	0.866	0.849
(iii) Negative affect (I-PANAS-SF)	5	0.882	0.894
3. Psychological Well-Being (Ryff’s PWBS)			
(i) Autonomy	2	0.749	0.704
(ii) Environmental mastery	2	0.695	0.736
(iii) Personal growth	2	0.799	0.883
(iv) Positive relation	2	0.670	0.675
(v) Self-acceptance	2	0.738	0.785
4. Perceived Stress (PSS)	10	0.799	0.831
5. Financial Desire Discrepancies (Brown’s FDD)			
(i) Desire discrepancy	2	0.806	0.725
(ii) Social desire discrepancy	2	0.819	0.809
(iii) Comparison desire discrepancy	2	0.829	0.755

**Table 4:** Results of Wilcoxon Signed-Rank Test.

Construct	Intervention Group (n = 95)			Control Group (n = 31)		
	Pre-Assessment	Post-Assessment	*p*-Value	Pre-Assessment	Post-Assessment	*p*-Value
	Mean (SD)	Mean (SD)		Mean (SD)	Mean (SD)	
Subjective Well-Being						
(i) Life satisfaction (SWLS)	4.02 (1.38)	4.36 (1.28)	0.006	3.77 (1.60)	4.04 (1.42)	0.361
(ii) Positive affect (I-PANAS-SF)	3.47 (0.79)	3.70 (0.74)	0.004	3.33 (0.71)	3.50 (0.80)	0.148
(iii) Negative affect (I-PANAS-SF)	2.42 (1.01)	2.41 (1.03)	0.456	2.64 (0.98)	2.90 (0.82)	0.151
Psychological Well-Being (Ryff’s PWBS)						
(i) Autonomy	4.68 (1.50)	5.10 (1.40)	0.012	5.28 (1.33)	4.84 (1.23)	0.060
(ii) Environmental mastery	4.96 (1.24)	5.17 (1.29)	0.038	5.3 (1.14)	5.04 (1.09)	0.293
(iii) Personal growth	6.07 (1.07)	6.26 (0.75)	0.089	6.31 (0.93)	5.97 (1.08)	0.206
(iv) Positive relation	4.24 (1.64)	4.59 (1.64)	0.019	4 (1.60)	4.13 (1.63)	0.654
(v) Self-acceptance	5.01 (1.36)	5.56 (0.96)	0	5.15 (1.40)	5.07 (1.12)	0.877
Perceived Stress (PSS)	2.05 (0.61)	1.93 (0.64)	0.031	2.11 (0.46)	2.04 (0.55)	0.361
Financial Desire Discrepancies (Brown’s FDD)						
(i) Desire discrepancies	4.38 (1.44)	3.94 (1.30)	0.001	4.6 (1.05)	4.17 (1.27)	0.116
(ii) Social desire discrepancy	3.97 (1.46)	3.77 (1.37)	0.097	4.2 (1.19)	4.13 (1.28)	0.565
(iii) Comparison desire discrepancy	3.24 (1.36)	3.24 (1.31)	0.467	3.7 (1.23)	3.57 (1.23)	0.528

**Table 5:** Descriptive Statistics of Intervention and Control Groups.

Items	Intervention Group (n = 95)		Control Group (n = 31)	
	Pre-Assessment	Post-Assessment	Pre-Assessment	Post-Assessment
	Mean (Std Dev)	Mean (Std Dev)	Mean (Std Dev)	Mean (Std Dev)
1. Mindfulness				
(i) Attention and awareness (MAAS)	4.01 (1.05)	4.14 (0.99)	3.77 (0.95)	3.59 (0.96)
2. Subjective Well-Being				
(i) Life satisfaction (SWLS)	4.02 (1.38)	4.36 (1.28)	3.76 (1.60)	4.04 (1.41)
(ii) Positive affect (I-PANAS-SF)	3.47 (0.79)	3.70 (0.74)	3.32 (0.71)	3.50 (0.80)
(iii) Negative affect (I-PANAS-SF)	2.42 (1.01)	2.41 (1.03)	2.64 (0.98)	2.90 (0.82)
3. Psychological Well-Being (Ryff’s PWBS)				
(i) Autonomy	4.69 (1.50)	5.10 (1.40)	5.27 (1.32)	4.84 (1.22)
(ii) Environmental mastery	4.96 (1.24)	5.17 (1.29)	5.29 (1.14)	5.03 (1.09)
(iii) Personal growth	6.07 (1.07)	6.26 (0.75)	6.31 (0.93)	5.97 (1.08)
(iv) Positive relation	4.24 (1.64)	4.59 (1.64)	4.00 (1.60)	4.13 (1.62)
(v) Self-acceptance	5.01 (1.36)	5.56 (0.96)	5.15 (1.39)	5.07 (1.12)
4. Perceived Stress (PSS)	2.05 (0.61)	1.93 (0.64)	2.11 (0.46)	2.04 (0.55)
5. Financial Desire Discrepancies (Brown’s FDD)				
(i) Desire discrepancy	4.38 (1.44)	3.94 (1.30)	4.60 (1.05)	4.16 (1.26)
(ii) Social desire discrepancy	3.97 (1.46)	3.77 (1.37)	4.19 (1.18)	4.13 (1.28)
(iii) Comparison desire discrepancy	3.24 (1.36)	3.24 (1.31)	3.69 (1.23)	3.57 (1.23)

**Table 6:** Composite Reliability and Average Variance Extracted.

Construct	Composite Reliability	Average Variance Extracted
Financial desire discrepancies	0.932	0.820
Subjective well-being	0.950	0.864
Mindfulness	0.935	0.673
Perceived stress	0.945	0.813
Psychological well-being	0.875	0.701

**Table 7:** Path Coefficients.

Hypothesis	Relationship	Std Beta	*t*-Values	*p*-Values
H1	Mindfulness -> Subjective Well-Being	0.162	1.348	0.089
H2	Mindfulness -> Psychological Well-Being	0.146	1.259	0.104
H3	Mindfulness -> Perceived Stress -> Subjective Well-Being	0.152	2.718	0.003
H4	Mindfulness -> Perceived Stress -> Psychological Well-Being	0.202	2.675	0.004
H5	Mindfulness -> Financial Desire Discrepancies -> Subjective Well-Being	0.035	0.738	0.230
H6	Mindfulness -> Financial Desire Discrepancies -> Psychological Well-Being	0.028	0.696	0.243

**Table 8:** PLS Predict (Structural Model Built vs. Linear Model).

Items	PLS RMSE	LM RMSE	PLS-LM
Subjective Well-Being			
Ideal life	1.548	1.623	−0.075
Satisfied with life	1.422	1.526	−0.104
Excellent life	1.365	1.490	−0.125
Psychological Well-Being			
Like own personality	0.969	0.978	−0.009
Pleased with how things turned out so far	1.055	1.054	0.001
Confident with own opinion	1.496	1.568	−0.072
Perceived Stress			
Nervous and stress	0.963	0.996	−0.043
Upset	0.961	0.987	−0.026
Unable to control things	0.991	1.050	−0.059
Felt difficulties	1.032	1.091	−0.059
Financial Desire Discrepancies			
What I have now vs. what I had 3 years ago	1.586	1.672	−0.086
What I have vs. what other people have	1.580	1.678	−0.098
What I have vs. what I want	1.662	1.746	−0.084

**Table 9:** Overall Findings (Hypotheses Testing).

Hypothesis	Relationship	Wilcoxon Signed-Rank Test	PLS-SEM	Results
		*p*-Value	*p*-Value	
H1	Mindfulness -> Subjective Well-Being	0.006	0.089	Supported
H2	Mindfulness -> Psychological Well-Being	0.000	0.104	Supported
H3	Mindfulness -> Perceived Stress -> Subjective Well-Being	n/a	0.003	Supported
H4	Mindfulness -> Perceived Stress -> Psychological Well-Being	n/a	0.004	Supported
H5	Mindfulness -> Financial Desire Discrepancies -> Subjective Well-Being	n/a	0.230	Not Supported
H6	Mindfulness -> Financial Desire Discrepancies -> Psychological Well-Being	n/a	0.243	Not Supported

**Figure A1:**
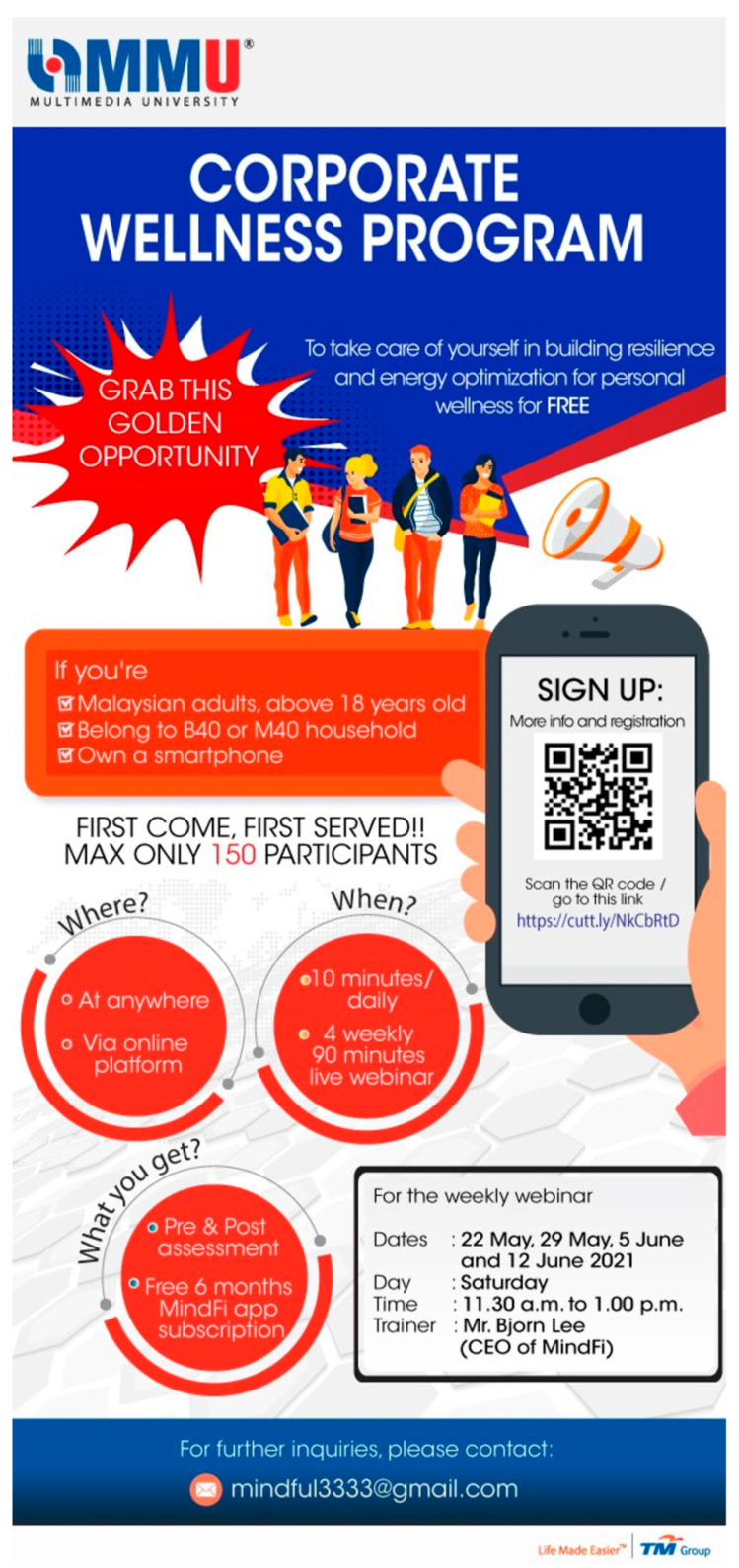
Recruitment Poster of Corporate Wellness Program.

## Data Availability

Not applicable.

## Appendix B

Items for Psychological Well-Being Scale in the questionnaire.

The answer format for all 18 items (1 = strongly agree; 2 = somewhat agree; 3 = a little agree; 4 = neither agree or disagree; 5 = a little disagree; 6 = somewhat disagree; 7 = strongly disagree)

There are 3 items for each construct as follow:

Construct: Autonomy

1. I tend to be influenced by people with strong opinions.
2. I have confidence in my own opinions, even if they are different from the way most other people think.
3. I judge myself by what I think is important, not by the values of what others think is important.

Construct: Environmental Mastery

1. The demands of everyday life often get me down.
2. In general, I feel I am in charge of the situation in which I live.
3. I am good at managing the responsibilities of daily life.

Construct: Personal Growth

1. For me, life has been a continuous process of learning, changing, and growth.
2. I think it is important to have new experiences that challenge how I think about myself and the world.
3. I gave up trying to make big improvements or changes in my life a long time ago.

Construct: Positive Relation

1. Maintaining close relationships has been difficult and frustrating for me.
2. People would describe me as a giving person, willing to share my time with others.
3. I have not experienced many warm and trusting relationships with others.

Construct: Purpose of Life

1. Some people wander aimlessly through life, but I am not one of them.
2. I live life one day at a time and don’t really think about the future.
3. I sometimes feel as if I’ve done all there is to do in life.

Construct: Self-Acceptance

1. I like most parts of my personality.
2. When I look at the story of my life, I am pleased with how things have turned out so far.
3. In many ways I feel disappointed about my achievements in life.

## Appendix C

Items Removed for the Analysis (Psychological Well-Being)

Construct: Autonomy

1. I tend to be influenced by people with strong opinions.

Construct: Environmental Mastery

1. The demands of everyday life often get me down.

Construct: Personal Growth

1. I gave up trying to make big improvements or changes in my life a long time ago.

Construct: Positive Relation

1. People would describe me as a giving person, willing to share my time with others.

Construct: Purpose of Life

1. Some people wander aimlessly through life, but I am not one of them.
2. I live life one day at a time and don’t really think about the future.
3. I sometimes feel as if I’ve done all there is to do in life.

Construct: Self-Acceptance

1. In many ways I feel disappointed about my achievements in life.
